# 2878. A Regional Antimicrobial Stewardship Collaboration to Address Fluoroquinolones

**DOI:** 10.1093/ofid/ofad500.155

**Published:** 2023-11-27

**Authors:** Ayne Adenew, Angela Y McKnight, Pawlose Ketema, Mirella Cole, Patricia Callahan, Ann Laake, Patrick Huffman, Rohini Dave, Jacqueline Bork, Ravi Tripathi, James Allman, Cecilia Gaynor, Travis Cork, Angelike P Liappis

**Affiliations:** DCVA Medical Center, washington, District of Columbia; DC VA Medical Center, Washington, District of Columbia; Veterans Affairs (VA) National Capitol Care Network (VISN5) Antimicrobial Stewardship Clinical Collaborative of VA Medical Centers in Washington DC/Martinsburg WV/Baltimore MD/Clarksburg WV/Beckley WV/Huntington WV, Washington, District of Columbia; Veterans Affairs (VA) National Capitol Care Network (VISN5) Antimicrobial Stewardship Clinical Collaborative of VA Medical Centers in Washington DC/Martinsburg WV/Baltimore MD/Clarksburg WV/Beckley WV/Huntington WV, Washington, District of Columbia; Veterans Affairs (VA) National Capitol Care Network (VISN5) Antimicrobial Stewardship Clinical Collaborative of VA Medical Centers in Washington DC/Martinsburg WV/Baltimore MD/Clarksburg WV/Beckley WV/Huntington WV, Washington, District of Columbia; Veterans Affairs (VA) National Capitol Care Network (VISN5) Antimicrobial Stewardship Clinical Collaborative of VA Medical Centers in Washington DC/Martinsburg WV/Baltimore MD/Clarksburg WV/Beckley WV/Huntington WV, Washington, District of Columbia; Veterans Affairs (VA) National Capitol Care Network (VISN5) Antimicrobial Stewardship Clinical Collaborative of VA Medical Centers in Washington DC/Martinsburg WV/Baltimore MD/Clarksburg WV/Beckley WV/Huntington WV, Washington, District of Columbia; VA Maryland Health Care System, Baltimore, MD; VA Maryland Health Care System, Baltimore, MD; Veterans Affairs (VA) National Capitol Care Network (VISN5) Antimicrobial Stewardship Clinical Collaborative of VA Medical Centers in Washington DC/Martinsburg WV/Baltimore MD/Clarksburg WV/Beckley WV/Huntington WV, Washington, District of Columbia; Veterans Affairs (VA) National Capitol Care Network (VISN5) Antimicrobial Stewardship Clinical Collaborative of VA Medical Centers in Washington DC/Martinsburg WV/Baltimore MD/Clarksburg WV/Beckley WV/Huntington WV, Washington, District of Columbia; Veterans Affairs (VA) National Capitol Care Network (VISN5) Antimicrobial Stewardship Clinical Collaborative of VA Medical Centers in Washington DC/Martinsburg WV/Baltimore MD/Clarksburg WV/Beckley WV/Huntington WV, Washington, District of Columbia; Veterans Affairs (VA) National Capitol Care Network (VISN5) Antimicrobial Stewardship Clinical Collaborative of VA Medical Centers in Washington DC/Martinsburg WV/Baltimore MD/Clarksburg WV/Beckley WV/Huntington WV, Washington, District of Columbia; Washington DC Veterans Affairs Medical Center, Washington, DC

## Abstract

**Background:**

Geographically associated Antimicrobial Stewardship Programs (ASPs) may benefit from working in collaboration. Independent ASPs can coordinate efforts with other programs to address high-yield targets for quality improvement and share in experience and knowledge to meet both local goals and fulfill national regulatory standards.

**Methods:**

In 2016 ASPs from six neighboring urban/rural VA Medical Centers serving 800K veterans agreed to target fluoroquinolone (FQ) use with shared messaging on preferred indications and reducing avoidable FQ toxicity. All sites had an established ASP and dedicated ASP Champions; two sites lacked FT ID trained MD and ASP FTE allocation varied per site. Sites pooled antibiograms 2018-22 and national metrics for FQ use in outpatient (OP) 2017-22, inpatient (IP) 2020-22 and the pandemic period (PP). Electronic platforms sustained communication and regular teleconferences facilitated planning and coordination. Local decisions determined interventions to target based on resources and patterns of use. FQ-targeted prospective audit/feedback, peer-peer reports and modified EMR- order entry were supplemented with shared educational materials. Messaging with reinforced with bi-annual regional events used targeted email campaigns and teleconferenced talks, including throughout the PP.

**Results:**

Over six years FQ total OP fills sustainably fell 13.6K to 4.3K with annual pharmacy visits 25K (1.5-7K site/year) and DOT/1,000 unique OP visits fell by nearly 70% in the same period across all sites urban/rural (P=.01). In the 522 (28-175) acute, 594 (50-275) LTC beds FQs fell 22.3 to 10.8 DOT/1,000 patient days by 2022. Regional-trends did not increase in PP, but decline slowed. In the region E. coli resistance was unchanged but in sites reporting FQs, suscepblity improved for isolates of MRSA (n 1294) and MSSA (n 1931); with sustained gains in the PP.

**Conclusion:**

Despite differences in hospital size, settings and resources, sustained outcomes can be achieved through efforts developed with regional partners. Coordinated regional stewardship initiatives facilitated by shared metrics are effective, support local efforts, allow for program autonomy and lessen the overall workload through shared experience.

**Disclosures:**

**All Authors**: No reported disclosures

